# The Impact of Pandemic Management on the Quality of Life of Slovak Dentists

**DOI:** 10.3390/ijerph18105484

**Published:** 2021-05-20

**Authors:** Veronika Pacutova, Andrea Madarasova Geckova, Peter Kizek, Andrea F. de Winter, Sijmen A. Reijneveld

**Affiliations:** 1 Department of Health Psychology and Research Methodology, Faculty of Medicine, P.J. Safarik University, 040 11 Kosice, Slovakia; andrea.geckova@upjs.sk; 2 Graduate School Kosice Institute for Society and Health, Faculty of Medicine, P.J. Safarik University in Kosice, 040 11 Kosice, Slovakia; 3 Olomouc University Social Health Institute, Palacky University, 775 15 Olomouc, Czech Republic; 4 Department of Community & Occupational Medicine, University Medical Center Groningen, University of Groningen, 9713 Groningen, The Netherlands; a.f.de.winter@umcg.nl (A.F.d.W.); s.a.reijneveld@umcg.nl (S.A.R.); 5 I. Stomatology clinic, University Hospital of Louis Pasteur, Faculty of Medicine, P.J. Safarik University, 040 11 Kosice, Slovakia; peter.kizek@unlp.sk

**Keywords:** COVID-19, pandemic management, quality of life, dentists, healthcare workers

## Abstract

Pandemic management increases the burden on healthcare workers to provide care and also affects their personal lives, with dentists being at particular risk. Therefore, we aim to describe the quality of life (QoL) and limitations experienced due to pandemic management-related measures (PanMan), as well as to assess the association of PanMan with QoL during the first lockdown after the coronavirus outbreak. We obtained data from 500 dentists (33.2% males, M/SD = 43.8) registered with the Slovak Chamber of Dentists using an online questionnaire. We categorized PanMan as the availability of personal protective equipment (PPE) and the ability to implement anti-pandemic measures, information overload, pandemic-related limitations and QoL in terms of their impact on family life and activities, housekeeping, relationships with relatives, financial situation and mental well-being. PanMan mainly affected financial situation, mental well-being and housekeeping. Factors contributing most towards the worsening of QoL were information overload (odds ratio/95% confidence interval, OR/CI: 5.79/2.64–12.71) and several pandemic-related limitations. These consisted of (OR/CI): a lack of PPE (5.17/2.48–10.77), infection risks in the work environment (3.06/1.57–5.95), obligatory safety measures (3.02/1.47–6.21), lack of staff (2.85/1.30–6.25) and client concerns (3.56/1.70–7.49). Pandemic management has led to a considerable worsening of dentists’ QoL.

## 1. Introduction

Coronavirus disease (COVID-19) and management of this pandemic had unprecedented consequences on the quality of life of healthcare workers (HCWs). Globally, COVID-19 has taken 2,659,802 lives as of 18 March 2021 [1], and in the first wave of the disease, before the possibility of being vaccinated, only pandemic management-related measures were in force to save human lives. Similar to other countries, Slovakia began to introduce early pandemic management (such as temperature screening, deep sanitation, closing schools and cancelling organized events) from the end of February 2020, and continued by enforcing the wearing of face masks, the closing of non-essential stores, a mandatory 14-day quarantine and home isolation. On 15 March 2020, Slovakia declared an “emergency healthcare situation” to provide the possibility of transferring healthcare workers from one hospital to another with no option of refusing to provide healthcare or declaring a strike [2]. However, pandemic management had already changed and affected the quality of life in physical, mental and social domains, both positively and negatively [3].

Healthcare workers were particularly vulnerable to the pandemic, but also to the side-effects of pandemic management-related measures. They stand on the frontline of a high-risk infectious work environment. Society also stigmatized HCWs and their relatives, with many members of the public avoiding them based on the belief that they could be a source of infection because of their profession and their contact with patients [4].

Dentists are a group of healthcare workers who had to drastically change their way of providing care during this outbreak. They typically have close contact with a patient’s mucosa, which could potentially be an easy way of virus transmission [5,6]. Pandemic management focused on dental care aimed to minimize the risk of infection by limiting the number of treatments, excluding preventive check-ups and dental hygiene from services and allowing only ”emergency cases” to be provided, with permitted hours from 8:00 a.m. until 12:00 p.m. Dentists were supposed to secure and wear personal protective equipment (FFP3/N95; closed glasses or full face shield; covering for the head, hands and legs; disposable gloves; shoe covers), and to maintain frequent disinfection with a reduced numbers of coworkers [7]. Both the pandemic and pandemic management-related measures are thus likely to have affected various aspects of the work and private lives of dentists, but evidence on this is fully lacking. Therefore, we aim to describe the quality of life (QoL) and limitations dentists experienced due to pandemic management-related measures (PanMan), as well as to assess the association of PanMan with QoL during the first lockdown after the coronavirus outbreak.

## 2. Materials and Methods

### 2.1. Sample

The study investigated all practicing dentists working in Slovakia (*n* = 3884), who were members of the Slovak Chamber of Dentists. These dentists were invited to participate in an online questionnaire from 8–12 October 2020; the invitation was sent by the administration of the Chamber to protect the privacy of the registered dentists. The questionnaire was specifically developed in cooperation with experts from the target population (Supplementary File). During multiple consultations with them, we elicited the relevant issue, drafted the questions, adjusted them based on their comments and piloted a draft final version to assure the clarity and appropriateness of the questionnaire. We received 635 responses, of which 15 participants had not filled in any of the questions. From those who filled in the questionnaire, we excluded those who did not report sex (*n* = 113) and those who were not dentists (2 nurses, 5 others); thus, the final sample consisted of 500 respondents (*n* = 166, 33.2% males, mean age/SD = 43.8/14.4).

### 2.2. Measures and Procedures

#### 2.2.1. Impact on the Quality of Life (QoL)

The impact on various fields of QoL was measured by asking respondents if difficulties in providing healthcare due to the introducing of pandemic management affected their (a) family life and activities, (b) housekeeping, (c) relationships with relatives, (d) financial situation and (e) mental well-being during the first wave of the COVID-19 pandemic. Answers on a Likert scale were dichotomized into those reporting worsening (slightly/significantly worsened) and improving (slightly/significantly improved vs. did not change) effects in a particular area of life.

#### 2.2.2. Impact of Pandemic Management-Related Measures (PanMan)

We measured PanMan as the availability of PPE and the ability to implement anti-pandemic measures, information overload and pandemic-related limitations. The availability of PPE covered whether respondents had all the necessary PPE at their disposal (personal contact with clients was excluded; were able/unable to fully assure protection; had to sometimes work in insufficiently protected conditions). The ability to implement anti-pandemic measures investigated how well respondents were able to implement and maintain anti-pandemic measures (unable/able to fully implement vs. unable to implement at all) during the first lockdown (March–June 2020). The following three groups of respondents were differentiated: not enough PPE and/or were unable to implement vs. enough PPE and able to implement fully protective measures. Information overload covered how frequently respondents followed news about the pandemic during the first lockdown (March–June 2020), and how much they were concerned about pandemic news. Combining these two questions, we divided the respondents into those who did not follow the news and/or were not concerned vs. those who followed the news several times per day and/or were highly concerned. Pandemic-related limitations of healthcare provision investigated (a) a lack of PPE, (b) infection risks in the work environment, (c) obligatory safety measures, (d) a lack of staff and (e) client concerns. Respondents were asked how much they were hindered in providing healthcare in its original quality during the first lockdown (March-June 2020). Answers on a Likert scale were dichotomized as partially/not limited vs. limited/significantly limited for each subcategory.

#### 2.2.3. Background Characteristics

We further measured the following background characteristics: gender, age and work position. 

### 2.3. Statistical Analysis

First, we described the background characteristics of the sample using descriptive statistics. Second, we described the QoL of dentists and the limitations experienced due to exposure to pandemic management-related measures, using prevalence data. Finally, we assessed the associations of PanMan (the availability of PPE and the ability to implement anti-pandemic measures, information overload, and pandemic-related limitations of the healthcare provided) with quality of life (family life and activities, housekeeping, relationships with relatives, financial situation, mental well-being) as separate outcomes, using logistic regression models with adjustment for gender and age. We used IBM SPSS Statistics 23 for Windows for preparing all analyses.

## 3. Results

### 3.1. Background Characteristics

The majority of dentists in our sample were owners of private dental clinics (77.8% of participants; *n* = 389), while 28.2% (*n* = 141) worked at private clinics and only 2.0% (*n* = 10) were from state dental clinics. Most of them were traditional dentists (86.2% of participants; *n* = 431) who treated adult and child clients (*n* = 467; 93.4%; Table 1).

### 3.2. Impact on Quality of Life and Exposure to PanMan

Most dentists reported that the pandemic has had an impact on their QoL, mostly regarding their financial situation and their mental well-being (85 and 70% of participants, respectively). The most frequently reported problems were those related to infection risks in their work environment and lack of PPE, reported by 63 and 55% of respondents respectively (Table 2).

### 3.3. Association of PanMan with QoL

Regarding QoL—family life, dentists who followed pandemic news several times per day and were concerned were more likely to report worsening effects on family life and activities (odds ratio/95% confidence interval, OR/CI: 1.71/1.02–2.88) than dentists who did not follow pandemic news. Findings are displayed in Table 3.

Regarding QoL—housekeeping, dentists who did not have enough PPE and were unable to implement anti-pandemic measures were more likely to report worsening effects on housekeeping (OR/CI: 1.88/1.11–3.20) than those whose had enough PPE and were able to implement anti-pandemic measures. Additionally, dentists who followed pandemic news several times per day and were concerned more frequently reported worsening effects on housekeeping (OR/CI: 2.58/1.51–4.40) than dentists who did not follow pandemic news. Moreover, those who reported that the care provided was limited/significantly limited due to a lack of PPE (OR/CI: 1.70/1.09–2.67), obligatory safety measures (OR/CI: 1.94/1.22–3.07) and a lack of staff (OR/CI: 1.89/1.18–3.01) were more likely to have felt worsening effects on housekeeping than those dentists who were partially/not limited.

Regarding QoL—relationships with relatives, dentists who reported that the care provided was limited/significantly limited due to a lack of staff (OR/CI: 2.78 (1.63–4.77) and client concerns (OR/CI: 2.09/1.23–3.54), were more likely to have felt worsening effects on their relationships with relatives than those dentists who were partially/not limited in their care.

Regarding QoL—financial situation, dentists who did not have enough PPE or/and were unable to implement anti-pandemic measures were more likely to report worsening effects on their financial situation (OR/CI: 2.41/1.16–5.01; OR/CI: 2.72/1.41–5.24) than those whose had enough PPE and were able to implement anti-pandemic measures. In addition, dentists who followed pandemic news several times per day and were concerned were more likely to report worsening effects on their financial situation (OR/CI: 2.78/1.04–7.46) than dentists who did not follow pandemic news. Moreover, those who reported that the care provided was limited/significantly limited due to a lack of PPE (OR/CI: 5.17/2.48–10.77), infection risks in the work environment (OR/CI: 3.06/1.57–5.95), obligatory safety measures (OR/CI: 3.02/1.47–6.21), a lack of staff (OR/CI: 2.85/1.30–6.25) and client concerns (OR/CI: 3.56/1.70–7.49), were more likely to feel worsening effects on their financial situation than those dentists who were partially/not limited.

Regarding QoL—mental well-being, dentists who did not have enough PPE and were unable to implement anti-pandemic measures were more likely to report worsening effects on their mental well-being (OR/CI: 2.15/1.26–3.68) than those who had enough PPE and were able to implement anti-pandemic measures. In addition, dentists who followed pandemic news several times per day and were concerned were more likely to report worsening effects on mental well-being (OR/CI: 5.79/2.64–12.71) than dentists who did not follow pandemic news. Moreover, those who reported that the care provided was limited/significantly limited due to a lack of PPE (OR/CI: 1.71/1.07–2.73) and reported infection risks in the work environment (OR/CI: 1.86/1.15–3.01), obligatory safety measures (OR/CI: 1.94/1.18–3.18), a lack of staff (2.41/1.40–4.16) and client concerns (OR/CI: 2.06/1.26–3.38) were more likely to feel worsening effects on mental well-being than those dentists who were partially/not limited.

## 4. Discussion

Dentists reported a poorer quality of life due to pandemic management-related measures aiming to prevent the spread of COVID-19 applied during lockdown, with more than 70% of them reporting worsening effects on both their financial situation and their mental health. In particular, some PanMan-related measures were felt to have been limiting, e.g., infection risks in work environment and a lack of PPE. PanMan in particular affected their financial situation, mental well-being and housekeeping.

We found the worst effects on QoL (85%) to be concerning financial situation, with the strongest associations concerning a lack of PPE and client concerns. The dentists’ financial situation was impacted most by the pandemic management-related measures that limited the number of treatments allowed [5]. Our data show that the number of patients dentists treated daily during lockdown dropped from 15–20 to less than 5. In addition, those who wanted to protect their family and not risk infection at work closed their clinics with financial concerns. Furthermore, on top of the reduced income, their expenditures increased due to mandatory personal protective equipment for healthcare workers, which were also highly overpriced at the beginning of the pandemic [6]. Dentists could not afford to stay unprotected and desperately needed to ensure PPE so that they would have the opportunity to provide safe care. Unfortunately, in some cases they were forced to reuse PPE, or work with badly fitting or no PPE [8,9,10]. Evidently, this impact on their finances also affected their mental well-being [11].

Mental well-being was the second most-severely affected domain of QoL, with the strongest associations concerning information overload and a lack of staff. All financial concerns, as well as limitations related to housekeeping, could affect the mental well-being of dentists, in whom a 70% increase in worsening effects was reported. COVID-19 and pandemic management measures could act as a stressor in their lives, and cause anxiety or depression [6,12,13,14]. Moreover, the required wearing of prescribed personal protective equipment led to discomfort and the development of skin problems, for example [15]. Other accompanying phenomena were panic, fear, exhaustion, anger, stigmatization or confusion [4,13]. This combination, along with the increased workload, also had the potential to lead to burnout or post-traumatic stress disorder [13,14,16,17,18,19,20], i.e., greatly affecting mental well-being. In other studies about healthcare workers’ QoL, the focus was mainly on nurses or physicians and their mental health. Burnout or secondary traumatization was associated with depression and anxiety, which caused an overall lower QoL [21,22,23,24]. Studies focusing on other healthcare professions, e.g., physiotherapists and optometrists, are still unpublished.

We found that 35% of all dentists reported a worsening of QoL, particularly in the field of housekeeping. The reason could be that a lack of proper information about the COVID-19 virus at the beginning of the pandemic coupled with the frequently changing pandemic management could have led to confusion and complication in the process of ensuring basic household supplements [25], household management and providing childcare. The increased workload of HCWs in general may have led to a decrease in time available for housekeeping [9], whereas this requires more time in general because of necessary cooking at home and coping with the possibility of closed stores. Furthermore, dentists who were parents had to take full responsibility for childcare and home-schooling, due to school closures and disappearing “helping hands” from grandparents, something which was required in order to protect the health of the elderly [26]. Gender differences may have contributed further, as most respondents were female and may have been taking care of the household and children more often [26,27].

PanMan had relatively less impact on QoL in the fields of family life and activities, as well as in relationships with relatives. An explanation may be that these relationships were relatively good, with the literature showing that even one strong supportive relationship could be enough to balance the impact of the pandemic [28]. Another explanation could be the wording of the question concerned, i.e., that respondents did not attribute worsened QoL to their ability to provide care. Evidently, this requires further study.

### 4.1. Strengths and Limitations

The main strength of our study is that we obtained a rather large national sample of dentists during the first wave of the COVID-19 pandemic. Moreover, we were able to gather information about the impact of a number of measures on a rather wide range of relevant QoL domains. However, some limitations should be considered. The major limitation may be the fact that the study is a picture of the situation during the first lockdown dentists ever encountered and is based on voluntary participation in an online survey. Results may thus have been affected by insufficient technological ability to complete the survey and could be different in the following waves of the COVID-19 outbreaks. Second, the online invitation for the survey via the Slovak Chamber of Dentists resulted in a relatively low response rate (12.9%), but the age and gender composition of our sample was quite similar to the target population, i.e., dentists registered in the Slovak Chamber of Dentists. There were slightly more female responses in our sample than the target population (66.8% vs. 61.4%), and fewer dentists over 66 years old (10.0% vs. 20.1%), the latter group being extremely small. Because of the adjustments we made for age and gender, this was unlikely to affect the findings. Third, the validity of the questions as they were asked deserves further confirmation. 

### 4.2. Implications

Our findings on the first pandemic situation in Slovakia show the importance of proper crisis management and anti-pandemic measures to ensure a safe work environment for dentists. The impact of anti-pandemic measures should continue to be assessed. Measures such as closing schools and kindergartens, stopping leisure activities for children and limitations in providing services create a large additional burden for families, in terms of managing their household and parenting. These measures should be balanced against their added value in curtailing the pandemic. Part of crisis management should be the provision of psychological support or peer support to dentists and their families, and similarly to other HCWs, support by governmental authorities. Dentists should not be forgotten amongst the pandemic essential care workers when mental support is to be offered. These findings require confirmation from other countries with other types of anti-pandemic measures.

## 5. Conclusions

Pandemic management has led to a considerable worsening of dentists’ QoL, in particular because of information overload and several pandemic-related limitations, i.e., a lack of PPE, infection risks in the work environment, obligatory safety measures, a lack of staff and client concerns.

## Figures and Tables

**Table 1:** Demographic characteristics of the respondents (Slovakia 2020; *n* = 500 dentists).

Variables	*n* (%)
**Sex:**	
Women	334 (66.8)
Men	166 (33.2)
**Type of dental clinic:**	
Private dental clinics—owners	389 (77.8)
Private dental clinics—employees	141 (28.2)
State dental clinics	10 (2.0)
**Specialization of dentists:**	
Traditional dentists	431 (86.2)
Specialized dentists	58 (11.6)
Other	11 (2.2)
**Type of patients treated:**	
Adults and children	467 (93.4)
Only adults	24 (4.8)
Only children	9 (1.8)

**Table 2:** Impact on quality of life (QoL) and exposure to effects of the pandemic management (PanMan) (Slovakia 2020; *n* = 500 dentists).

Variables	*n* (%)
**Slightly/Significantly worsened QoL in:**	
Family life and activities	190 (45.0)
Housekeeping	149 (35.4)
Relationships with relatives	89 (21.1)
Financial situation	362(85.4)
Mental well-being	298 (70.3)
**PanMan:**	
Availability of PPE and ability to implement anti-pandemic measures ^1^	222 (48.7)
Information overload ^2^	93 (19.6)
Pandemic-related limitation due to lack of PPE ^3^	200 (54.9)
Pandemic-related limitation due to infection risks in the work environment^3^	219 (62.6)
Pandemic-related limitation due to obligatory safety measures ^3^	169 (49.6)
Pandemic-related limitation due to lack of staff ^3^	131 (38.8)
Pandemic-related limitation due to client concerns ^3^	164 (47.1)

^1^ Not enough personal protective equipment (PPE) and unable to implement anti-pandemic measures; ^2^ followed the news several times per day and was highly concerned; ^3^ limited/significantly limited.

**Table 3:** The association of pandemic management with the quality of life of dentists; results of logistic regression analyses adjusted for age and gender leading to odds ratios, OR, and 95% confidence intervals (95%CI).

Variables	Family Life and Activities OR (95%CI)	Housekeeping OR (95%CI)	Relationships with Relatives OR (95%CI)	Financial Situation OR (95%CI)	Mental Well-Being OR (95%CI)
**Availability of PPE and ability to implement anti-pandemic measures**					
Enough PPE and able to implement	Ref	Ref	Ref	Ref	Ref
Not enough PPE or unable to implement	1.28 (0.74–2.23) ^ns^	1.13 (0.62–2.06) ^ns^	1.12 (0.55–2.31) ^ns^	2.41 (1.16–5.01) *	1.06 (0.60–1.87) ^ns^
Not enough PPE and unable to implement	1.46 (0.89–2.40) ^ns^	1.88 (1.11–3.20) *	1.73 (0.92–3.24) ^ns^	2.72 (1.41–5.24) **	2.15 (1.26–3.68) **
Age (in years)	1.01 (1.00–1.03) ^ns^	1.02 (1.00–1.03) *	1.01 (1.00–1.03) ^ns^	1.03 (1.01–1.06) **	1.01 (1.00–1.03) ^ns^
Male (vs. female)	1.05 (0.70–1.60) ^ns^	0.81 (0.52–1.26) ^ns^	0.88 (0.53–1.47) ^ns^	0.87 (0.49–1.57) ^ns^	0.67 (0.43–1.04) ^ns^
**Information overload**					
Did not follow, was not concerned	Ref	Ref	Ref	Ref	Ref
Followed the news or highly concerned	1.36 (0.87–2.13) ^ns^	1.37 (0.85–2.20) ^ns^	1.66 (0.97–2.85) ^ns^	0.96 (0.52–1.75) ^ns^	1.55 (0.96–2.50) ^ns^
Followed the news and highly concerned	1.71 (1.02–2.88) *	2.58 (1.51–4.40) ***	1.32 (0.70–2.49) ^ns^	2.78 (1.04–7.46) *	5.79 (2.64–12.71) ***
Age (in years)	1.01 (1.00–1.02) ^ns^	1.01 (1.00–1.02) ^ns^	1.01 (1.00–1.03) ^ns^	1.02 (1.00–1.04) *	1.00 (0.99–1.02) ^ns^
Male (vs. female)	1.09 (0.72–1.65) ^ns^	0.84 (0.54–1.31) ^ns^	0.90 (0.53–1.49) ^ns^	0.84 (0.47–1.50) ^ns^	0.71 (0.45–1.11) ^ns^
**Providing healthcare limited due to:**					
***Lack of PPE***					
Partially limited/ not limited	Ref	Ref	Ref	Ref	Ref
Limited/ Significantly limited	1.27 (0.83–1.94) ^ns^	1.70 (1.09–2.67) *	1.73 (1.02–2.92) ^ns^	5.17 (2.48–10.77) ***	1.71 (1.07–2.73) *
Age (in years)	1.01 (0.99–1.02) ^ns^	1.02 (1.00–1.03) *	1.01 (1.00–1.03) ^ns^	1.03 (1.01–1.06) **	1.02 (1.00–1.03) ^ns^
Male (vs. female)	1.22 (0.78–1.90) ^ns^	0.90 (0.56–1.44) ^ns^	1.11 (0.65–1.91) ^ns^	1.19 (0.59–2.39) ^ns^	0.76 (0.47–1.24) ^ns^
***Infection risks in the work environment***					
Partially limited/ not limited	Ref	Ref	Ref	Ref	Ref
Limited/ Significantly limited	1.07 (0.69–1.66) ^ns^	1.42 (0.89–2.27) ^ns^	1.33 (0.78–2.28) ^ns^	3.06 (1.57–5.95) ***	1.86 (1.15–3.01) *
Age (in years)	1.01 (0.99–1.02) ^ns^	1.02 (1.00–1.03) *	1.01 (1.00–1.03) ^ns^	1.03 (1.00–1.06) *	1.01 (1.00–1.03) ^ns^
Male (vs. female)	1.23 (0.78–1.94) ^ns^	0.91 (0.57–1.48) ^ns^	1.01 (0.58–1.73) ^ns^	1.07 (0.53–2.17) ^ns^	0.76 (0.46–1.25) ^ns^
***Obligatory safety measures***					
Partially limited/ not limited	Ref	Ref	Ref	Ref	Ref
Limited/ Significantly limited	1.00 (0.65–1.55) ^ns^	1.94 (1.22–3.07) **	1.66 (0.97–2.84) ^ns^	3.02 (1.47–6.21) **	1.94 (1.18–3.18) **
Age (in years)	1.01 (0.99–1.02) ^ns^	1.01 (1.00–1.03) ^ns^	1.01 (0.99–1.03) ^ns^	1.03 (1.00–1.05) *	1.01 (0.99–1.03) ^ns^
Male (vs. female)	1.18 (0.74–1.88) ^ns^	0.96 (0.58–1.57) ^ns^	1.12 (0.64–1.98) ^ns^	1.21 (0.60–2.45) ^ns^	0.82 (0.49–1.36) ^ns^
***Lack of staff***					
Partially limited/ not limited	Ref	Ref	Ref	Ref	Ref
Limited/ Significantly limited	1.14 (0.73–1.78) ^ns^	1.89 (1.18–3.01) **	2.78 (1.63–4.77) ***	2.85 (1.30–6.25) **	2.41 (1.40–4.16) **
Age (in years)	1.01 (0.99–1.02) ^ns^	1.02 (1.00–1.03) *	1.01 (1.00–1.03) ^ns^	1.03 (1.01–1.06) *	1.01 (1.00–1.03) ^ns^
Male (vs. female)	1.21 (0.76–1.93) ^ns^	0.91 (0.56–1.49) ^ns^	1.19 (0.67–2.10) ^ns^	1.04 (0.51–2.11) ^ns^	0.72 (0.43–1.20) ^ns^
***Client concerns***					
Partially limited/ not limited	Ref	Ref	Ref	Ref	Ref
Limited/ Significantly limited	0.99 (0.64–1.52) ^ns^	1.30 (0.83–2.04) ^ns^	2.09 (1.23–3.54) **	3.56 (1.70–7.49) ***	2.06 (1.26–3.38) **
Age (in years)	1.01 (0.99–1.02) ^ns^	1.02 (1.00–1.03) ^ns^	1.01 (1.00–1.03) ^ns^	1.04 (1.01–1.07) **	1.02 (1.00–1.03) ^ns^
Male (vs. female)	1.19 (0.75–1.87) ^ns^	0.84 (0.52–1.36) ^ns^	1.03 (0.59–1.79) ^ns^	0.97 (0.48–1.97) ^ns^	0.70 (0.43–1.16) ^ns^

* *p* < 0.05; ** *p* < 0.01; *** *p* < 0.001; ^ns^ – non significant; significant values in bold; Ref — reference value

## Data Availability

The data presented in this study are available on request from the corresponding author.

## Supplementary Materials

The following are available online at https://www.mdpi.com/article/10.3390/ijerph18105484/s1.
