# Supplementary material for: The Impact of Pandemic Management on the Quality of Life of Slovak Dentists
Source: Int J Environ Res Public Health. 2021 May 20;18(10):5484. doi: 10.3390/ijerph18105484 (PMC8161031; doi:10.3390/ijerph18105484)
Supplement: Supplementary file 1 [file ijerph-18-05484-s001.zip › ijerph-1169219-supplementary.pdf]

International Journal of
Environmental Research and Public Health

MDPI

Appendix A – COVID Questionnaire

Availability of PPE and ability to implement anti-pandemic measures:

Q.1 During the first lockdown, did you have at your disposal all the personal protective equipment needed?
- Personal contact with clients was excluded
- In personal contact with clients we were able to fully assure COVID protection
- In most contacts with clients we were unable to ensure sufficient protection
- During some periods we had to work with clients in insufficiently protected conditions

Q.2 How much were you able to implement and maintain COVID-related recommendations during the first lockdown?
- We were unable to implement them fully
- We were able to implement them fully
- We were unable to implement them at all

Information overload:

Q.1 Did you follow the news during the first lockdown?
- Yes, several times per day
- Yes, at least once a day
- Yes, but not more than usual
- No

Q.2 Were you concerned about the news?
- No
- A little concerned
- Very concerned

Providing healthcare limited due to:

Q.1 How much did the following circumstances hinder you in providing healthcare in the original quality?
(a, limited; b, significantly limited; c, partially limited; d, not limited)
- Lack of PPE
- Infection-risks in the work environment
- Obligatory safety measures
- Lack of staff
- Client concerns

Quality of Life:

Q.1 Did difficulties in providing healthcare due to introducing pandemic management affect:
(a, significantly improved; b, slightly improved; c, did not change; d, significantly worsen)
- Your family life and activities
- Your housekeeping
- Relationships with relatives
- Your financial situation
- Your mental well-being
